# Supplementary material for: Experimentally induced increases in fecundity lead to greater nestling care in blue tits
Source: Proc Biol Sci. 2019 Jun 26;286(1905):20191013. doi: 10.1098/rspb.2019.1013 (PMC6599988; doi:10.1098/rspb.2019.1013)
Supplement: Electronic supplementary material for Bründl et al. 2019 Proc Royal Soc B [file rspb20191013supp1.docx]

**Electronic supplementary material for Bründl et al. 2019 “Experimentally induced increases in fecundity lead to greater nestling care in blue tits”**

Authors: Aisha C. Bründl, Enrico Sorato, Louis Sallé, Alice C. Thiney, Sonja Kaulbarsch, Alexis S. Chaine, Andrew F. Russell

Journal: Proceedings of the Royal Society B: Biological Sciences

DOI: 10.1098/rspb.2019.1013

**Table S1A: Model summaries predicting female provisioning rate / h, from general linear models with Gaussian error structure and identity link function. The reference categories for treatment: experimental nests; for year: 2013.**

| Predictors |  | Estimate | ± SE | *F* value | *P* value |
| --- | --- | --- | --- | --- | --- |
| (Intercept) |  | 22.77 | 1.18 | 19.36 | <0.001 |
| Treatment | Control | -6.94 | 2.083 | 11.11 | <0.002 |
| Brood age |  | -0.010 | 0.76 | <0.001 | 0.99 |
| Brood age ^2 |  | -0.73 | 7.42 | 0.35 | 0.56 |
| Brood size |  | 1.58 | 0.61 | 6.64 | 0.013 |
| Brood size ^2 |  | -26.77 | 6.86 | 15.21 | <0.001 |
| Lay date |  | -0.41 | 0.35 | 0.24 | 0.63 |
| Altitude |  | 0.0050 | 0.0066 | 0.57 | 0.46 |
| Year | 2014 | -4.37 | 4.04 | 1.17 | 0.29 |
| Proportion of caterpillars |  | -9.8 | 6.61 | 2.2 | 0.15 |

**Table S1B:** **Model summaries predicting male provisioning rate / h, from general linear models with Gaussian error structure and identity link function. The reference categories for treatment: experimental nests; for year: 2013.**

| Predictors |  | Estimate | ± SE | *F* value | *P* value |
| --- | --- | --- | --- | --- | --- |
| (Intercept) |  | 111.19 | 22.72 | 4.89 | <0.001 |
| Treatment | Control | -2.98 | 1.76 | 2.86 | 0.098 |
| Brood age |  | 1.82 | 0.62 | 8.63 | <0.006 |
| Brood age ^2 |  | -0.68 | 7.023 | 0.0093 | 0.92 |
| Brood size |  | 1.38 | 0.57 | 5.95 | 0.019 |
| Brood size ^2 |  | -25.13 | 5.84 | 18.51 | <0.001 |
| Lay date |  | -1.0086 | 0.21 | 23.78 | <0.001 |
| Altitude |  | 0.0018 | 0.0082 | 0.047 | 0.83 |
| Year | 2014 | -9.39 | 2.47 | 14.41 | <0.001 |
| Proportion of caterpillars |  | -2.97 | 6.046 | 0.24 | 0.63 |

After the minimal model was determined we added parental age as a covariate (yearling versus older than 1-year) into the sex-specific provisioning models. The reason for this second step was that our sample size of parental age was smaller and thus, we did not want to reduce the power of our analysis of the general effect of treatment. In the 16 control nests we have information on 7 yearling females and 2 yearling males, and 5 older than 1-year females and 7 older than 1-year males. In the 34 experimental nests we have information on 17 yearling females and 9 yearling males, and 12 older than 1-year females and 10 older than 1-year males. Females provisioned at a higher rate with increasing age (LM: *F*_1,36_ = 12.15, *P* = <0.002); however, older males did not (LM: *F*_1,20_ = 1.43, *P* = 0.25). The treatment effect did not change significantly with age included in both models (females: LM: *F*_1,36_ = 18.76, *P* = <0.001; males: LM: *F*_1,20_ = 3.35, *P* = 0.082). There was no interaction between parental age and treatment (females: LM: *F*_1,35_ = 0.75, *P* = 0.39; males: LM: *F*_1,19_ = 0.11, *P* = 0.74). As such, the effect of age on feeding rate did not influence the effect of our experiment.

**Table S2A:** **Model summaries predicting total provisioning rate / h, from general linear models with Gaussian error structure and identity link function. The reference categories for treatment: experimental nests; for year: 2013.**

| Predictors |  | Estimate | ± SE | *F* value | *P* value |
| --- | --- | --- | --- | --- | --- |
| (Intercept) |  | 140.94 | 32.98 | 4.27 | <0.001 |
| Treatment | Control | -10.00 | 2.56 | 15.31 | <0.001 |
| Brood age |  | 1.92 | 0.90 | 4.56 | 0.038 |
| Brood age ^2 |  | -5.65 | 9.96 | 0.32 | 0.57 |
| Brood size |  | 3.023 | 0.94 | 10.32 | <0.003 |
| Brood size ^2 |  | -52.19 | 8.48 | 37.91 | <0.001 |
| Lay date |  | -1.085 | 0.30 | 13.061 | <0.001 |
| Altitude |  | 0.015 | 0.012 | 1.69 | 0.20 |
| Year | 2014 | -10.016 | 3.59 | 7.79 | <0.008 |

**Table S2B: Model summaries predicting chick mass, from linear mixed models with maximum likelihood (ML) estimation including nest box ID as random effect. The reference categories for treatment: experimental nests; for year: 2013.**

| Predictors |  | Estimate | ± SE | *χ2* value | *P* value |
| --- | --- | --- | --- | --- | --- |
| (Intercept) |  | 33.97 | 5.91 | 5.75 | <0.001 |
| Treatment | Control | -0.64 | 0.33 | 3.90 | 0.048 |
| Brood age |  | -0.56 | 0.29 | 3.90 | 0.048 |
| Brood size |  | 0.11 | 0.07 | 2.34 | 0.13 |
| Variance in hatching mass | | 0.77 | 0.50 | 2.46 | 0.12 |
| Lay date |  | -0.13 | 0.04 | 11.67 | <0.001 |
| Altitude |  | -0.0025 | 0.00092 | 7.84 | 0.005 |
| Year | 2014 | -0.24 | 0.70 | 0.12 | 0.73 |

**S1. Estimation of blue tit egg volume from photographs of clutches.**

Blue tit (*Cyanistes caeruleus*) egg volume was estimated using pictures of clutches taken in the field. Single egg images were subsequently extracted from clutch photographs using a semi-automated custom-built script, implemented in the image-processing software Fiji ImageJ (see below; [1]; <http://fiji.sc/Fiji>). Egg volume was then estimated within the *R* statistical environment by batch processing of single egg images using an image-processing custom-made script.

*R* code is available upon request (email: [enrico.srt@gmail.com](mailto:enrico.srt@gmail.com)). Below, we detail steps for egg volume estimations.

*Photographing clutches in the field*

Blue tit clutch photographs were taken following nest box inspections, between day 8 and 9 after the first egg was laid. Eggs were carefully removed from the nest and placed on a ca 15x10 cm cardboard grid, consisting of a holding board which was covered in 1 cm^2^ graph paper. Eggs were placed with their longest axis at a right angle to the camera. A piece of paper indicating nest identity and date was placed next to the eggs. Pictures of the grid with the eggs were then taken, using digital cameras (Canon IXUS 132, Canon PowerShot SX210 IS, Nikon COOLPIX L29, Nikon COOLPIX AW130 and Nikon D5100). Photographs (No. range =1 – 3 /clutch) were taken holding the camera perpendicular to the grid, between a distance of 30 to 40 cm, and by applying a 20-25 % magnifying zoom. Preliminary trials had indicated that this procedure minimize image distortion due to spherical aberration of the camera lenses.


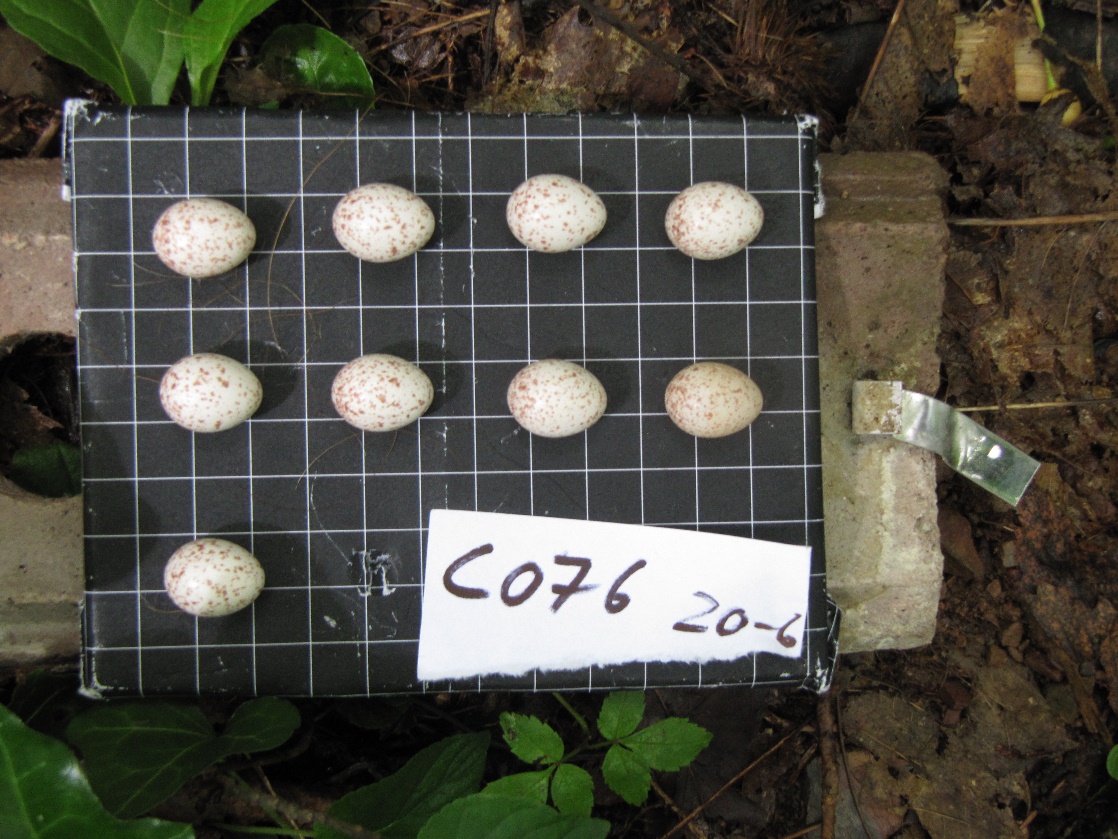


**Figure S1.** Photograph of a blue tit clutch. Each grid square has an area of 1 cm^2^ (1x1 cm)

*Egg extraction from digital camera pictures*

For each clutch, single egg-images were extracted from the best clutch photograph, i.e. from the picture yielding the sharpest definition of egg contours. Clutch pictures were opened in the image-processing software Fiji image-J, and single egg images were extracted by loading a custom-built macro. Briefly, within the macro, egg-contour was automatically identified by a shape-recognition plugin (“E-snake”; <http://bigwww.epfl.ch/algorithms/esnake/>), following manual selection of a rectangular ROI (“region of interest”) encompassing the egg to be extracted. The selected egg was then automatically saved as a fixed-size image with a black background. This procedure was sequentially repeated starting with the first egg in the picture (top left) and ending with the last one (bottom right). Hence, a clutch photograph of, e.g. six eggs, would produce six single-egg images, each named by the clutch ID, followed by a progressive egg number (1 to N, with N=clutch size). Following egg extraction, a random sample of three grid squares, in proximity of the eggs, was selected from the clutch picture, and the number of pixels contained in each of three 1 cm grid sides was counted. Average number of pixels corresponding to 1 cm was subsequently used to convert egg volume, measured in pixel units, into the corresponding volume in cm^3^.


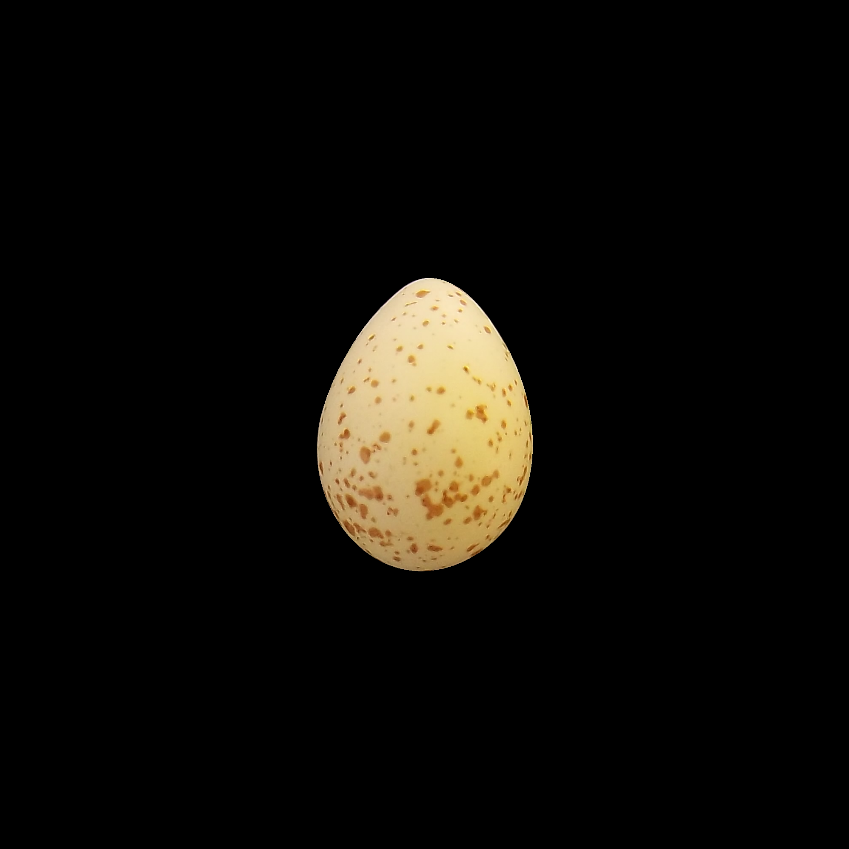

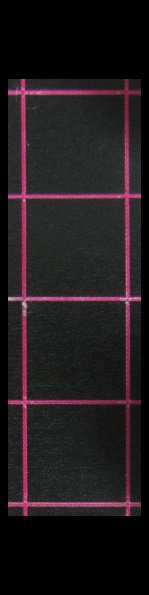


**Figure S2.** Example of extracted blue tit egg and grid squares sample, used for estimation of the conversion factor for egg volume calculation.

*Estimation of egg volume through image processing in R*

Egg volume was estimated from single-egg images within the *R* statistical environment using a custom-built image-processing script. First, egg images were imported into R, and binarized into black (background) and white (egg surface) images. Then, using image processing functions (R packages Biops [2] and EBImage [3]), the egg was rotated until its major axis was perpendicular to the x-axis (Fig. S3). Next, egg length and maximum width were calculated in pixel units. To estimate egg volume (pixel units), the egg surface image portion was sliced into sections of 1-pixel width, perpendicular to the major (vertical) egg axis; the volume of each corresponding egg disk slice was then calculated, assuming radial egg symmetry along it major axis, using the formula : V_slice_ = π*(d/2)^2^ wherein ‘d’ represented the width (along the x-axis), in pixels, of the egg slice. This procedure was repeated for each egg section and total egg volume was then calculated as the sum of the slices’ volumes. All eggs were batch processed using a loop that automatically imported and analysed egg images one by one, and saved results in an output table. Finally, egg volume measures were converted to cm^3^ units, using as a conversion factor the average number of pixels corresponding to a 1 cm grid square.


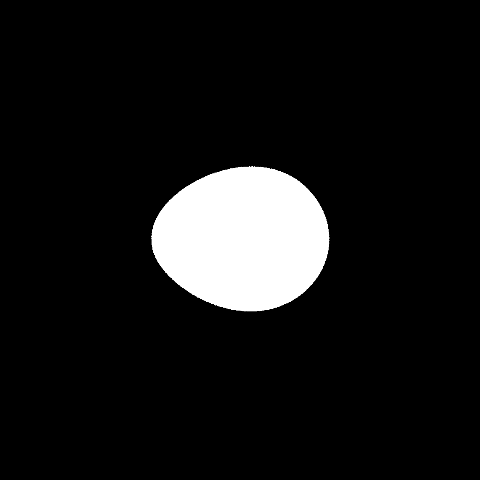


**Figure S3.** Example of a binarized egg image, used for egg volume estimation within the *R* statistical environment

*Validation*

In order to validate our egg volume estimation method, we estimated volume on a set of eight dummy plasticine eggs, of varying shape and size, analogous to the natural range observed in blue tit eggs (Fig. S4). First, egg length and maximum width were measured using callipers. Egg volume was then directly measured, using a graduate cylinder (2 cm diameter): the dummy egg was inserted into the cylinder, and entirely covered in sand. The total volume filled in the cylinder was then recorded (making sure the sand was evenly distributed). Next, the egg was removed from the cylinder, and volume of the sand measured. The difference between the two readings gave the egg volume.

Dummy eggs were subsequently photographed in the lab, using the same grid utilised for blue tit eggs, over a range of camera distances and zooming: pictures were taken at distance intervals of 10, 20, 30, 40, 50, 60 cm, overlapping with the range of camera distances for pictures of blue tit clutches. Further, at each distance, a picture was taken with and without zooming (with the exclusion of the 10 cm bin).


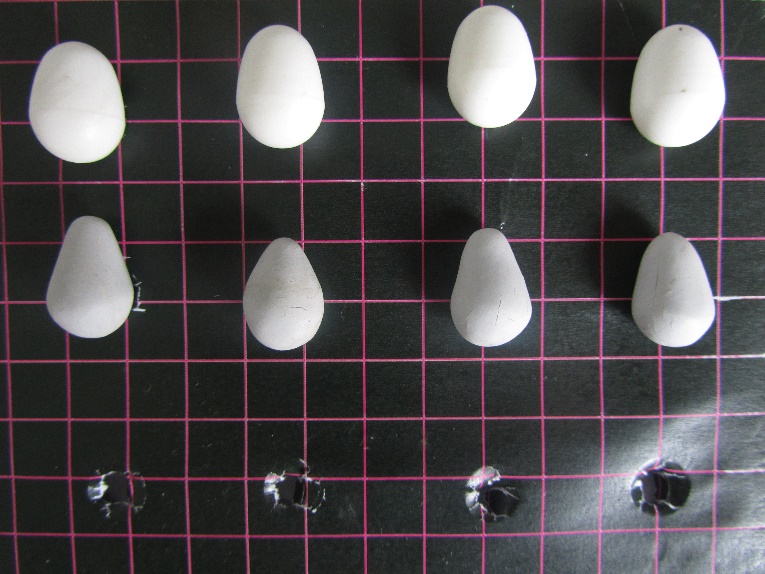


**Figure S4**. Dummy plasticine eggs of varying size and shape

Volume was then repeatedly estimated for each egg across the full range of camera distances and zooming, using the image processing method outlined above. In addition, we also calculated volume using the classical formula relating egg volume to egg length and diameter: 0.042+0.4673*egg length*egg breadth^2^ [4,5], with (i) length and diameter estimated from the picture (one estimate for each distance*zooming class), as well as (ii) using direct calliper measures.

Next, we modelled estimated egg volume as a function of camera distance and zooming, by conducting linear mixed models with egg identity as a random term, distance, zooming and their interaction as fixed effects, and a normal error distribution (R package lme4, function lmer, [6,7]). For egg volume estimated through the image processing method, we found a non-linear decrease in estimated mean-level volume as camera distance increased, particularly for picture taken without zooming (Fig. S5a); as expected, volume was overestimated at close distances (10-20 cm), owing to a higher percent difference in the distance of the camera from the background grid relative to the egg. Notably, at the 30 cm camera distance, estimates under zooming and no zooming conditions converged to the direct measure average value. Further, individual (egg) repeatability of volumes was very high (R = 0.91; Figure S5b), indicating that our image-based volume estimation method was effective in capturing the relative ranking in size across the dummy eggs.

Applying the same modelling approach to formula volume estimates from image-derived egg length and diameter measures, yielded the same relationship between volume estimate and distance*zooming conditions (Fig. S5c). However, volume was consistently underestimated, unless photographs were taken at close distances and without zooming. Repeatability of volume estimates was also lower compared to image-processing estimates (R= 0.81; Fig. S5d), suggesting that parametric formula approximations were less effective at estimating egg volume compared to our method.

(a)


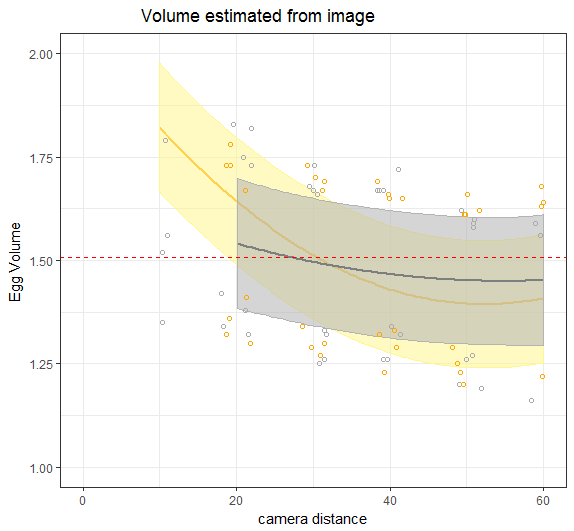

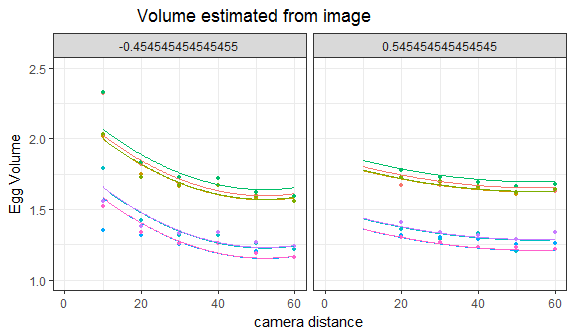


(b)

No zooming

Zooming

(d)


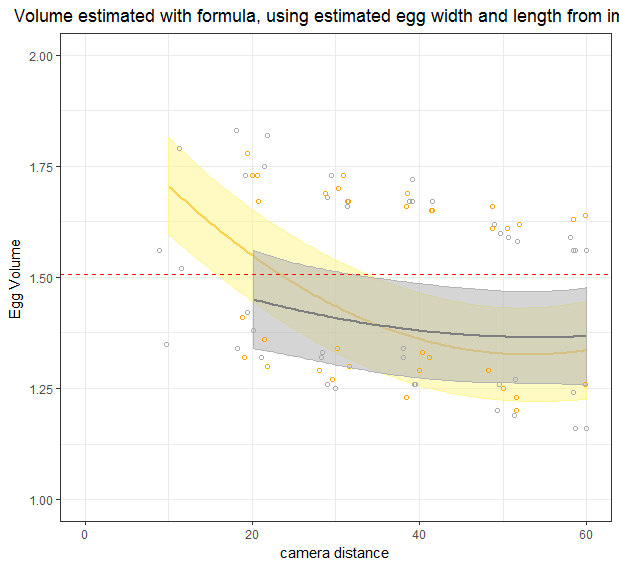


(c)


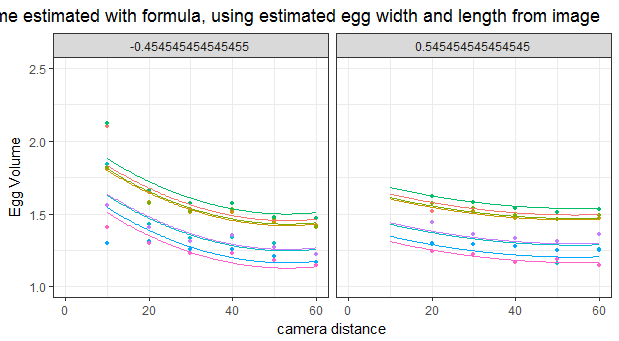


No zooming

Zooming

**Figure S5.** Relationship between estimated egg volume and camera distance x zooming. Top row: egg volume estimated through image processing in R; bottom row: formula volume, using picture-estimated egg length and width. Shown in (a) and (c) are mean-level estimates and corresponding confidence intervals (yellow: no zooming; grey: zooming); circles correspond to observed values, the red dashed horizontal line mark directly measured average egg volume. Shown in (b) and (d) are individual eggs estimates (random slopes predicted values) and corresponding observations.

As a further validation step, we calculated correlations between estimated volume and direct volume measures, across combinations of camera distances and zooming conditions. For the image-estimated volume, correlations were very strong across the entire range of distances * zooming conditions (all r > 0.93; Fig. S6a). Strong correlations were also found for formula estimates based on image-derived egg measures (all r > 0.87; Fig. S6b), though here biases leading to overestimation or underestimation were preponderant. Comparing formula estimates using calliper measures with direct volume measures also yielded a strong correlation (r=0.87), though this was weaker than for the previous two estimate types. In addition, inspection of the scatterplot suggest underestimation for large size eggs (Fig. S6c).

(a)


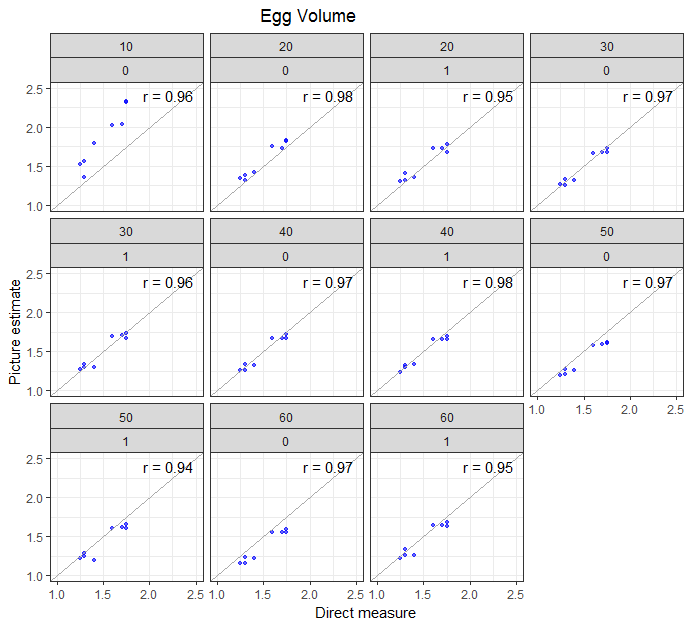

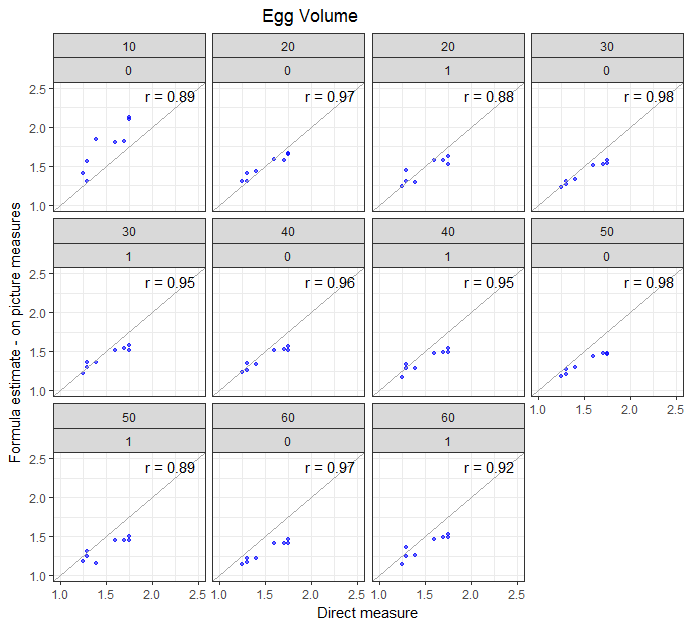


No zooming

No zooming

No zooming

No zooming

No zooming

No zooming

Zooming

Zooming

Zooming

Zooming

Zooming

No zooming

No zooming

No zooming

No zooming

No zooming

No zooming

Zooming

Zooming

Zooming

Zooming

Zooming

(b)

(c)


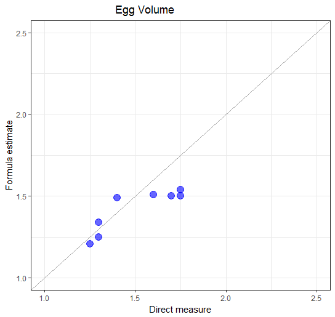


R = 0.86

Direct measure

**Figure S6.** Correlations between directly measured and estimated egg volumes. Panel (a) shows correlations using image-processing volume estimates, (b) correlations using formula calculations on egg length and width estimated from pictures; (c) correlations using formulas on calliper measures. The numbers after the panels in a) and b) refer to camera distances.

Finally, we assessed how the average bias in volume estimation (V_estimated_ – V_measured_) varied as a function of distance and zooming conditions, by visually inspecting plots of average bias and standard error (Fig. S7). Confirming previous results, bias was minimized by our image-based estimation method (Fig. S7a), with volume estimates matching direct measurements when pictures were taken at a distance of 30-40 cm. Conversely, the other two formula-based estimations were mostly underestimating volume (Fig. S7b,c).

(b)

(a)


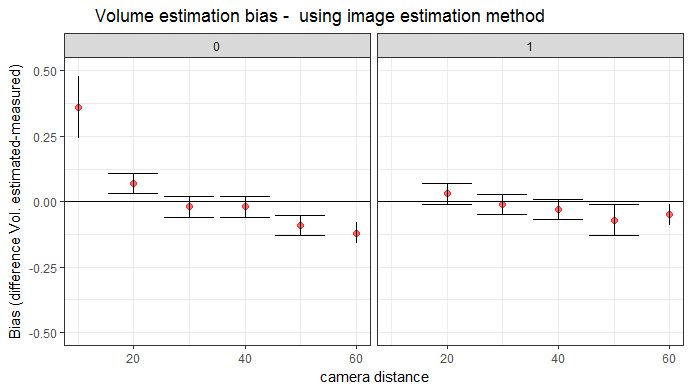

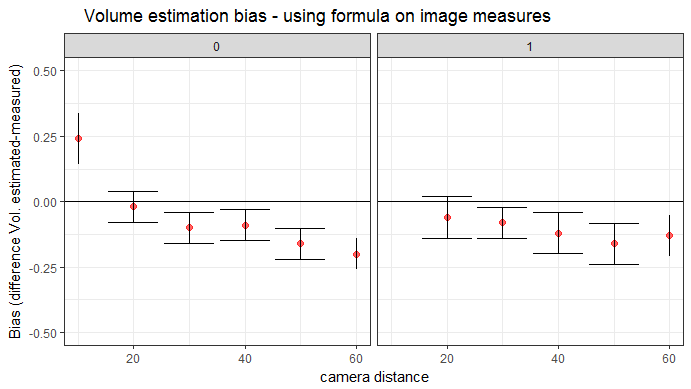

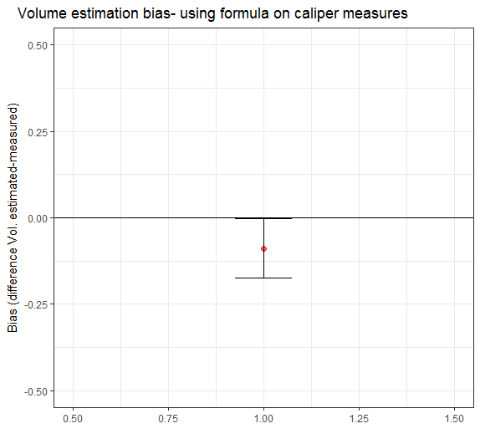


No zooming

No zooming

Zooming

Zooming

(c)

Camera distance

**Figure S7**. Estimation bias according to volume estimation method. Volume was estimated in (a) from egg pictures using image processing; in (b) using picture-estimated egg length and width; in (c) from formula on calliper measures. Red dots indicate average biases across the sample of eight dummy eggs; bars show 95 % confidence intervals.

Taken together, these validation results indicate that our image-based approach was superior to the formula-based method and provided unbiased estimates of egg volume over a range of camera distances coinciding with the protocol target for taking pictures of wild blue tits clutches. We therefore conclude that our estimates effectively captured variation in blue tit egg volume.

**References**

1. Schneider CA, Rasband WS, Eliceiri KW. 2012 NIH Image to ImageJ: 25 years of image analysis. *Nat. Methods* **9**, 671–675.

2. Bordese M, Alini W. 2013 *biOps: Image processing and analysis*. See http://www2.uaem.mx/r-mirror/web/packages/biOps/index.html.

3. Pau G, Fuchs F, Sklyar O, Boutros M, Huber W. 2010 EBImage—an R package for image processing with applications to cellular phenotypes. *Bioinformatics* **26**, 979–981.

4. Ojanen M, Orell M, Väisänen RA. 1978 Egg and clutch sizes in four passerine species in northern Finland. *Ornis Fenn* **55**, 60–68.

5. Bańbura M, Glądalski M, Kaliński A, Markowski M, Skwarska J, Wawrzyniak J, Zieliński P, Bańbura J. 2018 A consistent long-lasting pattern of spatial variation in egg size and shape in blue tits (*Cyanistes caeruleus*). *Front. Zool.* **15**, 34.

6. Bolker BM, Brooks ME, Clark CJ, Geange SW, Poulsen JR, Stevens MHH, White J-SS. 2009 Generalized linear mixed models: a practical guide for ecology and evolution. *Trends Ecol. Evol.* **24**, 127–135.

7. Bates D, Mächler M, Bolker B, Walker S. 2015 Fitting linear mixed-effects models using lme4. *J. Stat. Softw.* , 6442.
